# Eco-Virological Patterns of Zoonotic Viruses in Neotropical Bats: Insights for Host Species Distributed in the Northern Andes—A Scoping Review and Trait-Based Analysis

**DOI:** 10.3390/v18101148

**Published:** 2026-10-16

**Authors:** J. Milena Astorquiza, Leonardo Padilla Sanabria, Laura Ávila-Vargas, Julián Ruiz-Saenz

**Affiliations:** 1 Molecular Immunology Group (GYMOL), Universidad del Quindío, Avenida Bolívar 12N-001, Armenia 630004, Colombia; lpadilla@uniquindio.edu.co; 2 Evolutionary Ecology Group (GIEE), Universidad de Nariño, Ciudad Universitaria Torobajo, Calle 18 Carrera 50, Pasto 520002, Colombia; 3 One Health Research Group, Faculty of Agricultural Sciences, Universidad de La Salle, Bogotá 110811, Colombia; 4 Grupo de Investigación en Ciencias Animales—GRICA, Facultad de Medicina Veterinaria y Zootecnia, Universidad Cooperativa de Colombia, Bucaramanga 680001, Colombia

**Keywords:** chiroptera, Neotropics, Northern Andes, ecological traits, host–virus associations, One Health

## Abstract

Bats are recognized as important reservoirs of zoonotic viruses; however, the ecological determinants shaping virus–host associations in regions of high biodiversity remain poorly understood. In this study, we conducted a scoping review and a trait-based analysis to characterize eco-virological patterns of zoonotic RNA viruses reported in Neotropical bat species whose distributions include the Northern Andes (Colombia, Ecuador, and Venezuela). We compiled 578 unique viral detection records reported across Neotropics from 235 studies and sequence records for 56 bat species occurring in the Northern Andes, including members of the families *Coronaviridae*, *Flaviviridae*, *Orthomyxoviridae*, *Paramyxoviridae*, and *Rhabdoviridae*. Ecological, spatial, and life-history traits of host species were incorporated, and their associations were evaluated using multiple correspondence analysis, chi-square tests, and Cramér’s V statistics. Although multivariate analyses and flow diagrams suggested recurrent ecological patterns, statistical tests revealed weak and non-significant associations between viral families and ecological traits (V = 0.05–0.20; *p* > 0.05). Viral records were concentrated in lowland, non-migratory species with generalist roosting behavior and frequent use of anthropogenic habitats; however, these patterns likely reflect host availability and sampling effort rather than ecological specialization. Viral detections were distributed across multiple ecological contexts, suggesting broad virus–host associations among Neotropical bat species. Importantly, we identified a marked geographical bias in the available evidence: 64.0% of records originated from Brazil, whereas direct records from the Northern Andes were scarce (Colombia: 5.0%) or absent (Ecuador and Venezuela: 0%). Consequently, our findings provide a baseline of potential viral diversity and virus–host associations for bat species occurring in the Northern Andes rather than direct evidence of local viral circulation. These results highlight critical knowledge gaps and underscore the need for strengthened surveillance and One Health research across the Northern Andes.

## 1. Introduction

Bats, known for their remarkable diversity and ecological roles, are globally recognized as natural reservoirs of a wide range of zoonotic viruses [1]. Their unique immune systems, high mobility, and social behaviors facilitate the persistence and transmission of these [2,3]. Collectively, these biological characteristics contribute to the dynamics of viral maintenance in bat communities and increase opportunities for cross-species transmission [1,4].

In this context, the increasing frequency of viral spillover events from bats to other species, including humans, makes it crucial to elucidate the ecopathological and environmental factors that facilitate such transmission [2,3,4,5,6]. This is particularly relevant in Neotropical regions such as the Andes, where the highest taxonomic diversity of bats has been documented, with a significant concentration of species in the Andean region of Colombia [7,8].

It has been predicted that in regions with high biodiversity, the interaction between reservoirs and susceptible hosts can intensify the risk of zoonotic emergencies [6,9] given that these regions concentrate a high diversity of viruses [10]. This pattern is partly related to the dilution hypothesis, according to which host diversity can buffer or amplify pathogen transmission depending on the composition of the ecological community [11,12].

Previous research has primarily focused on identifying specific viral families and host species, often overlooking the complex ecological and spatial dimensions of these dynamic [13,14]. While some studies have linked viral presence to bat phylogeny or diet, the role of broader landscape-level changes and a comprehensive set of natural history traits remains poorly understood [13]. In particular, the fragmentation of natural habitats and the expansion of urban and rural settlements create new interfaces where humans, domestic animals, and wildlife interact, which could increase the risk of viral spread [15,16,17]. However, it remains unclear how these ecological and landscape contexts influence the prevalence of zoonotic viruses in bat communities [15].

This review addresses this knowledge gap by evaluating how ecological, spatial, and natural history traits may modulate virus–host associations in Neotropical bat species, using the Northern Andes as a focal region of interest. We integrated a scoping review and a trait-based analytical framework to explore potential virus–host associations in bat species whose distributions include the Northern Andes. This region was selected because it harbors exceptional bat diversity and has experienced extensive anthropogenic transformation, resulting in increasingly close interactions at the human–animal interface [18,19].

In this context, the primary objective of this study was to analyze a comprehensive Neotropical dataset of viral detection records reported for bat species whose geographic distributions include the Northern Andes (Colombia, Ecuador, and Venezuela), to explore potential virus–host associations and identify critical regional information gaps. Specifically, we compiled detection reports of potentially zoonotic RNA viruses belonging to the families *Coronaviridae*, *Flaviviridae*, *Orthomyxoviridae*, *Paramyxoviridae*, and *Rhabdoviridae* reported across the Neotropics for 56 bat species occurring in the Northern Andes. Additionally, we evaluated host ecological traits (spatial patterns, habitat use intensity, and life-history traits) to characterize ecological patterns of virus–host associations and establish a baseline for future surveillance, One Health research, and conservation planning in this highly biodiverse yet understudied region.

## 2. Materials and Methods

### 2.1. Data Collection

Considering the increasing anthropogenic transformation of ecosystems in the Northern Andes and the recognized role of bats as reservoirs of viruses with zoonotic relevance, a scoping review was conducted to compile records of RNA viruses belonging to the families *Coronaviridae*, *Flaviviridae*, *Orthomyxoviridae*, *Paramyxoviridae*, and *Rhabdoviridae* reported across the Neotropics in bat species whose geographic distributions include the Northern Andes (Colombia, Ecuador, and Venezuela), following Preferred Reporting Items for Systematic Reviews and Meta-Analyses extension for Scoping Reviews (PRISMA-ScR) guidelines and the Joanna Briggs Institute framework [20,21]. The scoping review was registered at https://doi.org/10.17605/OSF.IO/U5MGA (accessed on 23 September 2026).

These viral families were selected because they include some of the most frequently reported RNA viruses in Neotropical bats [22] and encompass taxa with documented zoonotic transmission, demonstrated spillover capacity, or recognized potential for cross-species transmission among mammals [23]. Specifically, *Flaviviridae* was included due to the well-documented circulation of arboviruses in the Neotropics [24,25]; *Rhabdoviridae* because of the long-standing rabies surveillance systems established across the region [26,27]; *Paramyxoviridae* owing to its relatively high detection frequency in surveillance studies of Neotropical bats [28]; and *Orthomyxoviridae* because of the high diversity of wildlife hosts in the region and recent reports of bat-associated influenza A viruses in South America [29,30,31]. Other RNA viral families with recognized zoonotic potential in mammals, including *Arenaviridae*, *Filoviridae*, and *Bunyaviridae* [6], were not included in the present review because they remain only sparsely documented in Neotropical bats. The scope was therefore intentionally restricted to the five viral families listed above to focus the synthesis on well-documented RNA virus groups and to facilitate consistent comparisons among studies. Consequently, the diversity patterns described herein should be interpreted within the limits of the selected viral families. For the purposes of this review, viruses were classified as zoonotic or potentially zoonotic when previous evidence demonstrated infection in humans or other non-chiropteran mammals, or when experimental and epidemiological evidence indicated the potential for host switching, following the criteria proposed by Olival et al. [6].

The reviewed articles were obtained from the databases of the National Center for Biotechnology Information (NCBI) of the National Library of Medicine (MedLine/PubMed; http://www.ncbi.nlm.nih.gov/pubmed) and Web of Science (https://www.webofscience.com/. The access date for all databases was 22 December 2025. Additionally, available sequences in GenBank were consulted through the NCBI platform (https://www.ncbi.nlm.nih.gov/nucleotide/, accessed on 22 December 2025) and in The Database of Zoonotic and Vector-Borne Viruses (ZOVER https://www.mgc.ac.cn/cgi-bin/ZOVER/main.cgi, accessed on 22 December 2025).

From the selected studies, a database was constructed including sequence accession number (when available), host family, bat species, viral family, viral taxon, sampling location, authors, and study reference (Supplementary Material S1). For this review, a viral record was defined as a unique virus–host association reported for a given bat species within an individual study. Multiple detections of the same viral taxon in the same host species within a study were consolidated into a single record, whereas detections involving different viral taxa, host species, or independent studies were treated as separate records. This procedure was implemented to minimize duplicate observations and reduce potential duplicity from repeated detections of the same virus–host association within individual studies. Only records supported by molecular evidence, including polymerase chain reaction (PCR), sequencing, or metagenomic approaches, were retained.

### 2.2. Search Strategy

For each search in the PubMed and Web of Science databases, a phrase composed of different parts joined by the Boolean operators AND and OR was used. The search strategy was not limited to study design or language, and no specific year restrictions were established. All review and opinion articles on viral infections, publications such as notes, and conference abstracts, as well as articles on serological diagnosis or referencing other animal species or countries outside the Neotropics, were manually excluded. In all databases, the following search strategies were used: “bat OR bats OR Chiroptera” AND “virus OR zoonotic virus OR zoonosis” AND “America* OR Neotropical OR South America OR Central America”. The search syntax was adapted to the indexing structure and controlled vocabulary of each database when necessary.

*Inclusion Criteria*—Original studies reporting the molecular detection (PCR, sequencing, or metagenomic approaches) of viruses belonging to the families *Coronaviridae*, *Flaviviridae*, *Orthomyxoviridae*, *Paramyxoviridae*, and/or *Rhabdoviridae* reported across the Neotropics were considered. To be included, studies had to provide clear information regarding host species identification, sample type, and molecular diagnostic methodology, specifically targeting bat species whose geographic distributions encompass the Northern Andes (Colombia, Ecuador, and Venezuela). Articles published in indexed scientific journals and available in full text were retained.

*Exclusion Criteria*—Studies based exclusively on serological evidence were excluded because antibody detection does not confirm active viral infection or recent viral circulation. In addition, serological assays may be affected by cross-reactivity among antigenically related viruses, potentially generating uncertainty in viral identification and host–virus associations [32]. Reviews, opinion articles, reports without full-text access, and studies that did not clearly specify the viral detection methodology for the viral families under study were also excluded. Additionally, studies that did not allow confirmation of the presence of viruses from the families under study, reported bat species whose geographic distributions do not include the Northern Andes (Colombia, Ecuador, and Venezuela), or presented insufficient information about host identity or sample origin were discarded.

A two-phase approach was used for article selection. During the first phase, articles were selected based on their titles and abstracts, excluding those deemed irrelevant to the topic. In the second phase, the full text of each article included in the first phase was reviewed. Articles that did not meet the pre-established criteria were excluded. To ensure methodological robustness, article selection and data cleaning were conducted independently by two researchers, and all discrepancies were resolved through discussion with the research team.

### 2.3. Ecological, Spatial, and Life-History Traits of Bat Hosts

For complementing information on virus–host associations, an additional database was constructed integrating ecological, spatial, and natural history traits of bat species reported as hosts of the viral families included in this review. The database was structured at the species level with distribution in the Northern Andes region. It included information from specialized literature, biodiversity repositories, and reference sources, including the International Union for Conservation of Nature (IUCN) Red List (https://www.iucnredlist.org/, accessed on 22 December 2025), biodiversity databases (Global Biodiversity Information Facility (GBIF), https://www.gbif.org/es/, accessed on 22 December 2025, Biodiversity Information System of Colombia (SiB Colombia), https://biodiversidad.co/, accessed on 22 December 2025), and a review of relevant specialized literature on regional distribution, including documentation published in Mammals of South America [33].

For each species, ten variables were compiled that, according to documented scientific evidence, may influence host–virus associations and the dynamics of zoonotic viruses [6,34]. Whenever available, ecological and spatial information was extracted directly from the metadata reported in the original studies included in the review. To complement missing information, additional data were obtained from specialized literature, biodiversity repositories, and species accounts describing the natural history and ecological characteristics of each bat species. These variables were grouped into three categories defined from the scientific literature: (i) spatial and movement patterns; (ii) habitat use intensity; and (iii) life history traits and social organization.

*Spatial and movement patterns*—This category included three variables: elevation, migratory behavior, and shelter type. The elevation documented at viral recording sites for each bat species was subclassified considering the thermal floors and life zones characteristic of the tropical Andes, suggested by Espinoza et al. [35] and Holdridge [36], establishing the following variables: Low elevation (0–1000 masl), corresponding to the warm and humid lowlands of foothills and inter-Andean valleys; medium elevation (1101–2100 masl), encompassing the lower and middle montane forest, with temperate temperatures; and high elevation (>2100 masl), which includes the upper montane forest and páramo, with cold conditions. These thresholds also align with significant changes in the composition of the mammalian community in the Andean region [37] and Neotropical frugivorous bats [38].

In the variable Migration, the displacement of each species was considered in two mutually exclusive categories, following the terminology and descriptions established in the work documented by Gardner [33] and supplemented with specific literature on bats movements, subclassified as: Migratory species, those that exhibit regular seasonal movement patterns along an elevation gradient, typically between low and high areas in response to climatically seasonal resource availability (e.g., fruiting, insect abundance). This subcategory also considered those species that undertake latitudinal and/or longitudinal movements over long distances with minimal elevation change, often associated with continental climatic patterns. In non-migratory species, we included species for which no regular seasonal long-distance movements are documented and whose home range remains relatively constant throughout the year.

The assignment to either of these two categories was based on explicit descriptions in the sources. For species with contradictory or ambiguous information, the conservative criterion of classifying them as “Non-Migratory” was adopted unless there was strong and repeated evidence of seasonal movements.

For the assignment of the shelter type variable, the species roosting or resting habits were considered, following the guidelines suggested by Frick et al. [39] and Jung & Threlfall [40], subclassifying the variable into one of the following mutually exclusive categories: Natural Shelter, referring to species that predominantly rely on non-anthropogenic structures for resting, such as caves, rock crevices, tree cavities, dense foliage, or modified leaves (e.g., tent-makers). Artificial Shelter, for species that predominantly frequent human-built structures, such as buildings, bridges, tunnels, house roofs, or urban infrastructure. Generalist or Facultative, for species that have been documented to regularly use both types of shelters (natural and artificial) without a clear preference in the reviewed literature.

When there were conflicting reports, priority was given to information from studies in the Andean region. This tripartite division (Natural, Artificial, Generalist) reflects the spectrum of synanthropy in bats and is a widely used criterion to assess their response to habitat disturbance and their potential for interaction with human environments as proposed by Frick et al. [39], Jung & Threlfall [40], and Kunz & Fenton [41].

*Habitat Use Intensity*—Three types of habitats were documented, primarily following the habitat selection criteria for Neotropical bats (e.g., Cisneros et al. [42]; Farneda et al. [43]; García-Morales et al. [44]; Meyer et al. [45]). These include: Natural Areas (primary forests, secondary forests, páramos, natural savannas); Agricultural Areas (crops, pastures, plantations, agricultural mosaics); and Anthropogenic Areas (urban, peri-urban, industrial, and densely modified rural zones). For each species and habitat type, an association level was assigned based on the frequency with which the species was reported in that habitat in the reviewed literature, following a standardized criterion: (i) Very Frequent (high): The species was described as common, abundant, or typical of the habitat, or was reported in this category in over 60% of the sources mentioning its habitat; (ii) Frequent (medium): The species was described as present or recorded in the habitat but not dominant, or was reported under this category in 30% to 60% of relevant sources; and (iii) Rare (low): The species was described as occasional, accidental, or sporadically recorded in the habitat, or was reported in less than 30% of sources. When a species was reported in various habitats with the same frequency, multiple categories were assigned.

*Life history traits and social organization*—This category included four variables: trophic guild, reproductive cycle, size, and colony type. In the trophic guild, we included the functional classification established for Neotropical bats (e.g., Aguirre et al. [46]; Kalko & Handley [47]; Sampaio et al. [48]), categorizing them into one of the five main guilds: Frugivore, Insectivore, Nectarivore, Omnivore, or Hematophage. This variable serves as a proxy for trophic position and resources exploited, factors that influence exposure to environmental pathogens and microbiome composition [6,34].

Regarding the reproductive cycle, we classified the species based on the documented patterns for Neotropical bats [33]. Based on the premise that the estrous cycle in chiropterans is characterized by the sequence of stages between estrus periods, defined as the phase when the female accepts copulation [49], and that it is frequently synchronized with resource availability such as flowering and fruiting [50]. Three categories were established: Continuous Polyestrous, species with two or more estrous cycles per year, which can be continuous or interrupted by periods of anestrus (reproductive inactivity) [49,51,52]. Seasonal Polyestrous, reproductive activity with two or more estrous cycles strictly regulated by environmental factors (e.g., photoperiod, rainfall) that determine specific windows for reproduction [52]. Seasonal Monoestrous, species with a single annual estrous cycle, typically associated with a season of high resource availability [51,52].

This variable is epidemiologically crucial, as seasonality and reproductive synchrony can modulate population dynamics (creating cohorts of susceptible juveniles) and immune physiology, generating temporal windows of increased risk for the exacerbation of infections or the vertical transmission of pathogens [4,53].

The social variables involving colony size and type were subcategorized considering the aggregation structure documented for Neotropical bats [41,54]. Given the broad ecological diversity of the study species, ranging from solitary to highly gregarious in colony size, the following operational classification was established, adapted from criteria used in studies of Neotropical bat assemblages [33,54,55,56]: Small colony: Up to 50 individuals, including solitary species with temporary shelters and/or family groups, typical of non-gregarious species (e.g., species of the genus *Sturnira*, *Dermanura*, *Rhinophylla*). Medium colony: From 51 to fewer than 500 individuals, mainly including gregarious species that form colonies in shelters such as caves, hollow trees, and buildings (e.g., species of the genus *Carollia*, *Artibeus*, *Glossophaga*, *Desmodus*). Large colony: More than 500 individuals. Characteristic of species with a high tendency to form large assemblies in extensive shelters (e.g., *Phyllostomus hastatus*, *Molossus* spp., *Tadarida brasiliensis*). It is important to note that the category was assigned based on the maximum size reported in the literature for the region.

Regarding the type of association or colony in the shelter, it was classified according to the information documented by Gardner [33], particularly for Neotropical bats, and mainly considering three groupings: Conspecific colonies, when species group with individuals of the same species; Congeneric colonies, when species share colonies with other species of the same taxonomic genus; and Mixed colonies, for species that group with other species.

### 2.4. Statistical Analysis

*Limitations of data design*—The constructed database (Supplementary Material S1) is based on evidence available in the literature and is therefore subject to detection bias. Bat species were included only when at least one molecularly confirmed record of a virus belonging to the target viral families was available. This implies that the analyzed set represents a group of positive species from a viral perspective, and not a random or complete sample of bats distributed in the Andes. Consequently, the statistical analyses describe patterns of association within this group, and their results should be interpreted as the ecological characterization of species that have been identified as carriers, rather than as absolute predictors of viral risk across the entire bat community.

*Multiple Correspondence Analysis (MCA)*—To explore patterns of association among viral families and host ecological traits, a Multiple Correspondence Analysis (MCA) was performed using a matrix composed exclusively of categorical variables. The analysis included the five viral families (*Coronaviridae*, *Flaviviridae*, *Orthomyxoviridae*, *Paramyxoviridae*, and *Rhabdoviridae*) together with all ecological variables described in Supplementary Material S2 (Ecology analysis), including elevation, migratory behavior, roost type, habitat-use intensity (natural, agricultural, and anthropogenic areas), trophic guild, reproductive cycle, colony size, and colony type. MCA was used as an exploratory ordination technique to summarize the relationships among multiple categorical variables in a reduced-dimensional space. The analysis was conducted using the FactoMineR package, and graphical visualization of category coordinates was generated using the factoextra, ggplot2, and ggrepel packages; all analyses were performed in R version 4.5.1. Category positions in the ordination space were interpreted based on their relative proximity, where categories located closer together indicate a higher tendency to co-occur within the dataset, whereas categories located farther apart indicate weaker associations. Full eigenvalues, percentage of inertia per dimension, and category contributions are reported in Supplementary Material S3.

*Alluvial diagrams*—To visually summarize the distribution and co-occurrence of viral families and ecological trait categories, alluvial diagrams were generated using the ggsankey package in R (version 4.5.1, R Foundation for Statistical Computing, Vienna, Austria). Prior to visualization, records were grouped according to unique combinations of viral families and ecological trait categories, and the frequency of each combination was calculated. The width of the flows was proportional to the number of records sharing the same combination of categories. These diagrams were used exclusively as an exploratory and descriptive visualization tool to illustrate patterns of category co-occurrence across variables and were not intended to provide statistical evidence of association.

*Chi-square tests of independence and association strength*—To evaluate whether viral families were associated with host ecological traits, contingency tables were constructed from the frequency of records in each category and analyzed separately for each ecological variable. The null hypothesis stated that viral family occurrence was independent of the ecological trait category, whereas the alternative hypothesis stated that an association existed between both variables. Analyses were carried out in R (version 4.5.1) using the chisq.test() function from the *stats* package. For each contingency table, the Chi-square statistic, degrees of freedom, and associated *p*-value were calculated, and statistical significance was assessed at α = 0.05. Because several viral families contained relatively few records distributed across multiple ecological categories, expected cell frequencies were evaluated for each contingency table. When more than 20% of cells had expected frequencies below five, the Chi-square approximation was considered unreliable and Fisher’s exact test with Monte Carlo simulation (10,000 replicates) was used as the inferential test for that variable. To quantify the strength of associations, Cramér’s V was calculated using the cramerV() function from the *rcompanion* package. Given that no universally standardized threshold exists for interpreting Cramér’s V in ecological or biological contexts [57,58] effect sizes were reported directly rather than assigned fixed categorical labels and were interpreted cautiously in light of the sample size underlying each contingency table.

## 3. Results

### 3.1. Overview of the Included Records

The initial systematic search yielded a total of 1630 articles, with a higher representation in PubMed (*n* = 1090) compared to Web of Science (*n* = 540). After the filtering process, 214 scientific articles were selected, which, along with the integration of 21 viral sequences from GenBank (NCBI—Nucleotide) not linked to scientific articles, consolidated a total of 235 studies. From this set of information, 578 unique virus records belonging to the families *Coronaviridae*, *Flaviviridae*, *Orthomyxoviridae*, *Paramyxoviridae*, and *Rhabdoviridae*, linked to 56 bat species present in the Northern Andes, were identified.

**Figure 1:**
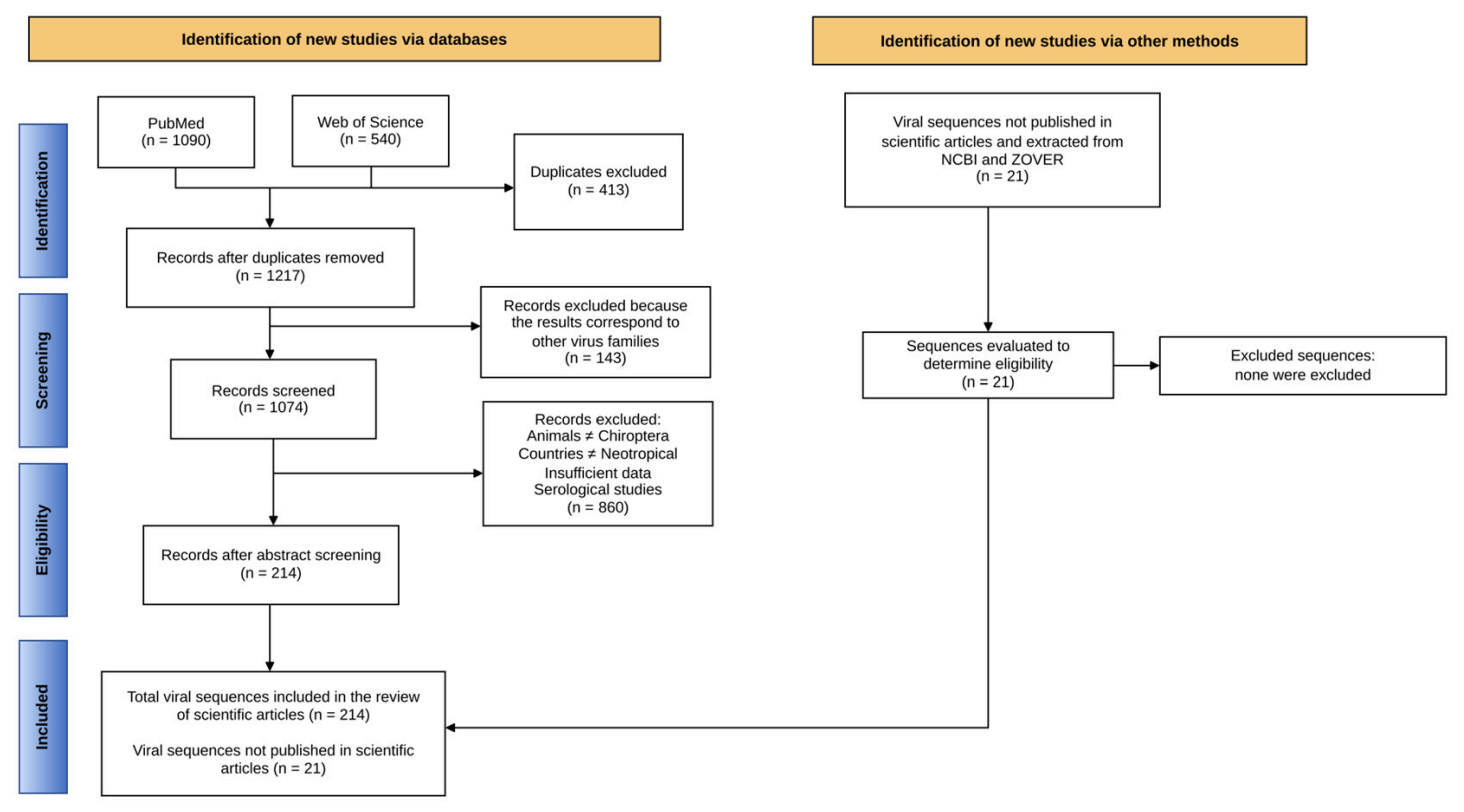
Preferred Reporting Items for Systematic Reviews and Meta-Analyses (PRISMA) flow diagram of the literature search and study selection process.

It is worth noting that these records include detections reported in the Neotropics, allowing us to infer the viral diversity associated with these hosts in the region. Regarding the origin of the information, 96.4% (*n* = 557) comes from formally published studies, while the remaining 3.6% (*n* = 21) corresponded to sequences that have not yet been published in scientific literature (Figure 1; Supplementary Material S1).

### 3.2. Geographical Distribution of the Records

**Figure 2:**
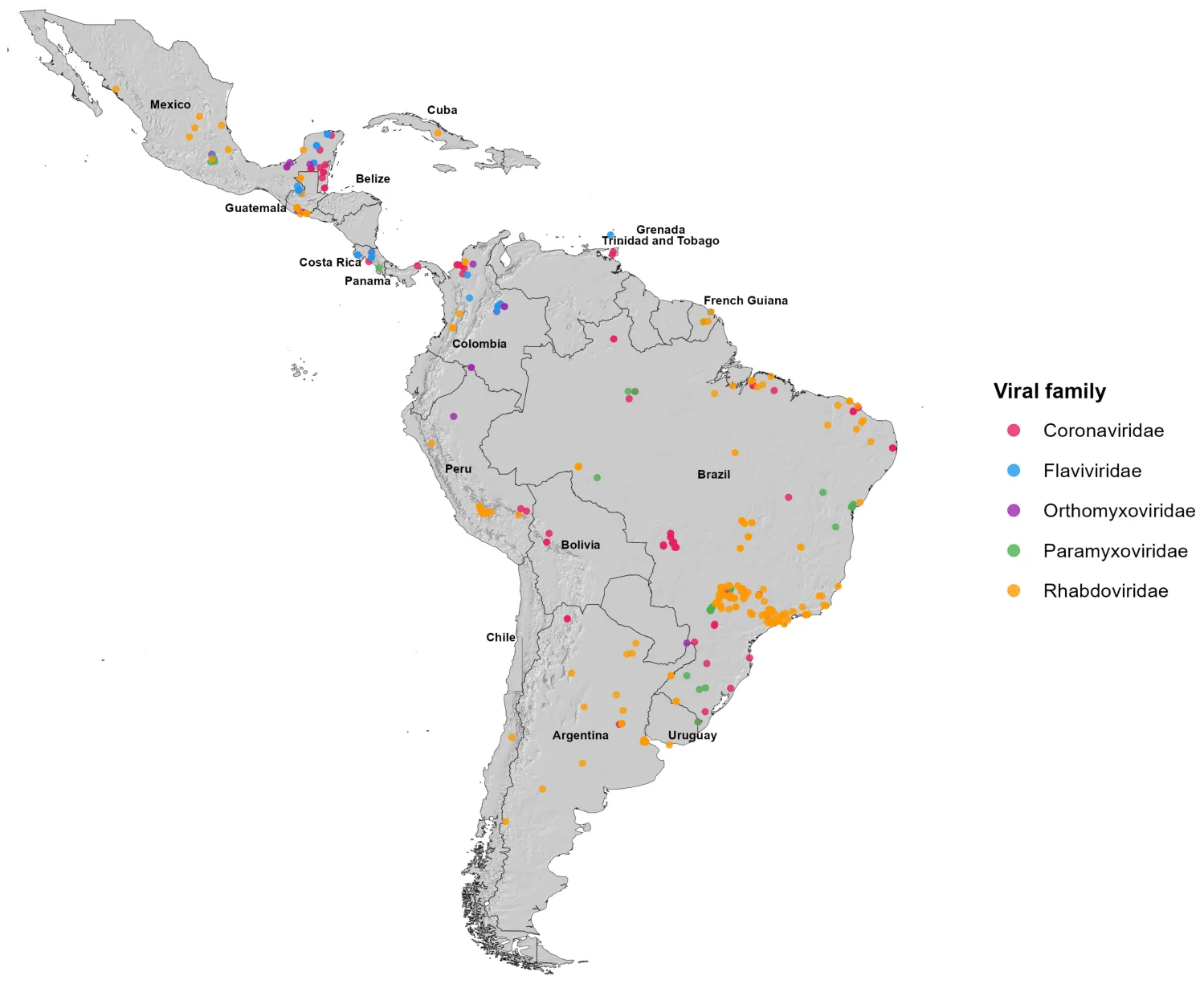
Geographical distribution of viral records in the Neotropics associated with bat species identified as potential hosts in the Northern Andes.

The records associated with hosts distributed in the Northern Andes were distributed across 16 Neotropical countries, showing marked heterogeneity in sampling effort and viral detection frequency (Figure 2). Brazil accounted for the largest proportion of records (*n* = 370; 64.0%), followed by Mexico (*n* = 55; 9.5%), Argentina (*n* = 37; 6.4%), Colombia (*n* = 29; 5.0%), and Peru (*n* = 27; 4.7%). In contrast, the contribution of countries such as Cuba, Chile, Grenada, Panama, and Trinidad and Tobago was considerably lower. Additionally, the absence of records in some Central American countries, as well as in Ecuador, Paraguay, and Venezuela, is notable (Figure 2).

The distribution of viral families among countries was heterogeneous. Records of *Rhabdoviridae* predominated in Brazil (*n* = 251), Argentina (*n* = 32), Peru (*n* = 18), and Mexico (*n* = 17). Colombia and Guatemala exhibited a comparatively higher representation of *Flaviviridae* (*n* = 6 in both countries) and *Orthomyxoviridae* (Colombia: *n* = 7; Guatemala: *n* = 2). Records of *Paramyxoviridae* were primarily concentrated in Brazil (*n* = 28) and French Guiana (*n* = 2) (Figure 2; Supplementary Material S1).

### 3.3. Taxonomic Distribution of Bat Hosts and Associated Viral Diversity

**Figure 3:**
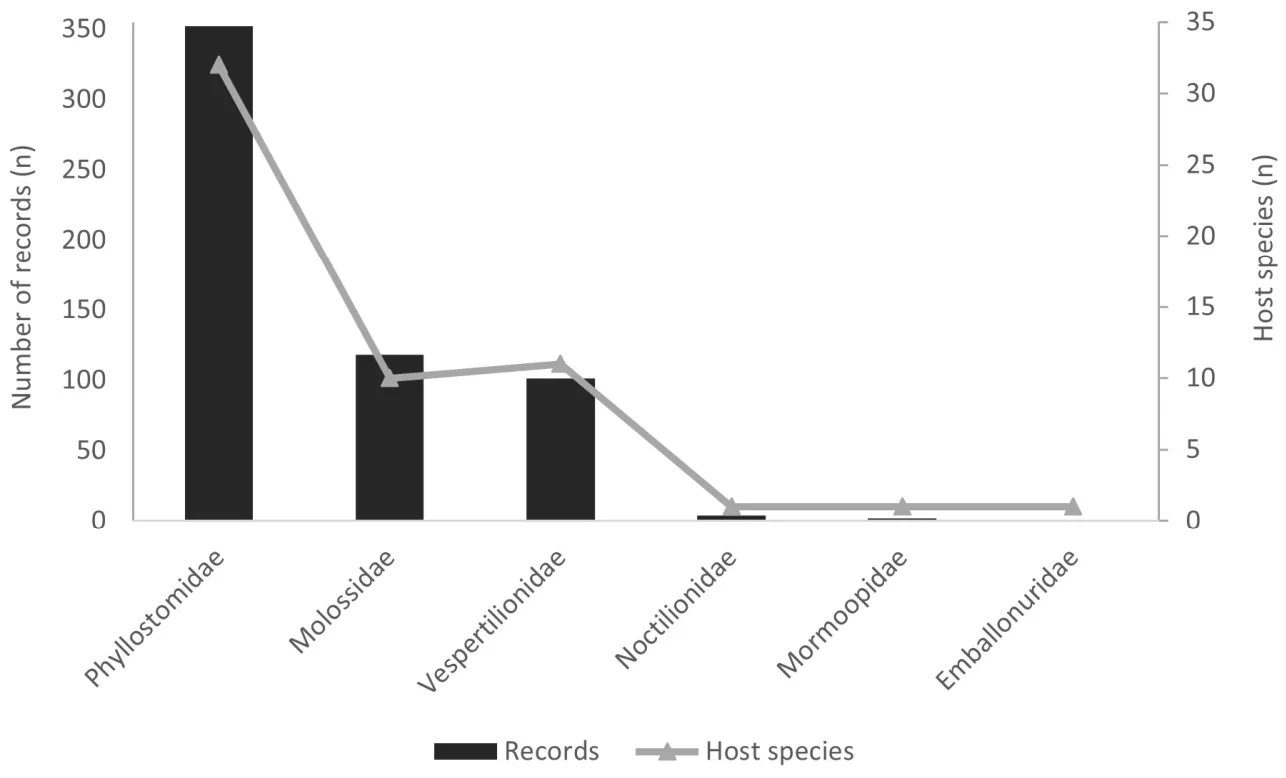
Records and species of bats potentially hosting in the Northern Andes region.

The 578 identified records corresponded to six bat families (Figure 3), with a clear predominance of the Phyllostomidae family. This family accounted for 60.9% of the records (*n* = 352), distributed across 32 species, and was also notable as the only family present in the five analyzed viral families (Figure 3). The Molossidae (20.4%; *n* = 118; 10 species) and Vespertilionidae (17.5%; *n* = 101; 11 species) families showed intermediate representation. In contrast, the Noctilionidae, Mormoopidae, and Emballonuridae families had marginal participation, collectively totaling seven records from three species (Figure 3).

Regarding the associations between host families and viral families, Phyllostomidae recorded the highest richness of interactions: *Rhabdoviridae* (*n* = 166), *Coronaviridae* (*n* = 107), *Flaviviridae* (*n* = 36), *Paramyxoviridae* (*n* = 30), and *Orthomyxoviridae* (*n* = 13). Molossidae and Vespertilionidae showed a pattern dominated by *Rhabdoviridae*, with 91 and 93 records respectively, followed by *Coronaviridae* in both families (*n* = 20 and *n* = 6). The families Noctilionidae, Mormoopidae, and Emballonuridae showed very limited representation, between one and four records in total (Table 1).

### 3.4. Representativeness and Taxonomic Diversity of the Identified Viral Families

Regarding the viral composition, a marked heterogeneity was observed both in the frequency of records and in the diversity of the taxa identified (Table 2). It is noteworthy that the family *Rhabdoviridae* had the highest representation with 352 records, all of which corresponded to a single species: *Lyssavirus rabies*. In contrast, *Coronaviridae* (*n* = 136) showed a distribution primarily centered in the genus *Alphacoronavirus*, which accounted for 74% of its records (*n* = 101), followed by *Betacoronavirus* (*n* = 8) and a group of records that remained with limited taxonomic resolution at the subfamily or family level (*n* = 27).

On the other hand, the family *Flaviviridae* (*n* = 43) was distinguished by presenting the greatest taxonomic diversity with seven identified taxa, among which *Orthoflavivirus denguei* (*n* = 16) and some species of *Pegivirus* (*n* = 10) and *Hepacivirus* (*n* = 4) stand out.

In the case of *Paramyxoviridae* (*n* = 33), six taxonomic levels were identified; however, a considerable portion of their records remained unclassified at the family level (*n* = 14), while the rest were linked to genera such as *Parajeilongvirus* (*n* = 10) and *Parajeilongvirus* (*n* = 8). Finally, *Orthomyxoviridae* was the least represented family (*n* = 14), being restricted exclusively to the virus Influenza A (*n* = 10) and its avian lineage subtypes H17N10 and H18N11 (Table 2). Taxonomic resolution varied considerably across viral families, ranging from single-species assignment in *Rhabdoviridae* to multiple co-occurring taxa with partial resolution in *Flaviviridae* and *Paramyxoviridae*.

### 3.5. Ecological Analysis and Virus–Host Association Patterns

**Figure 4:**
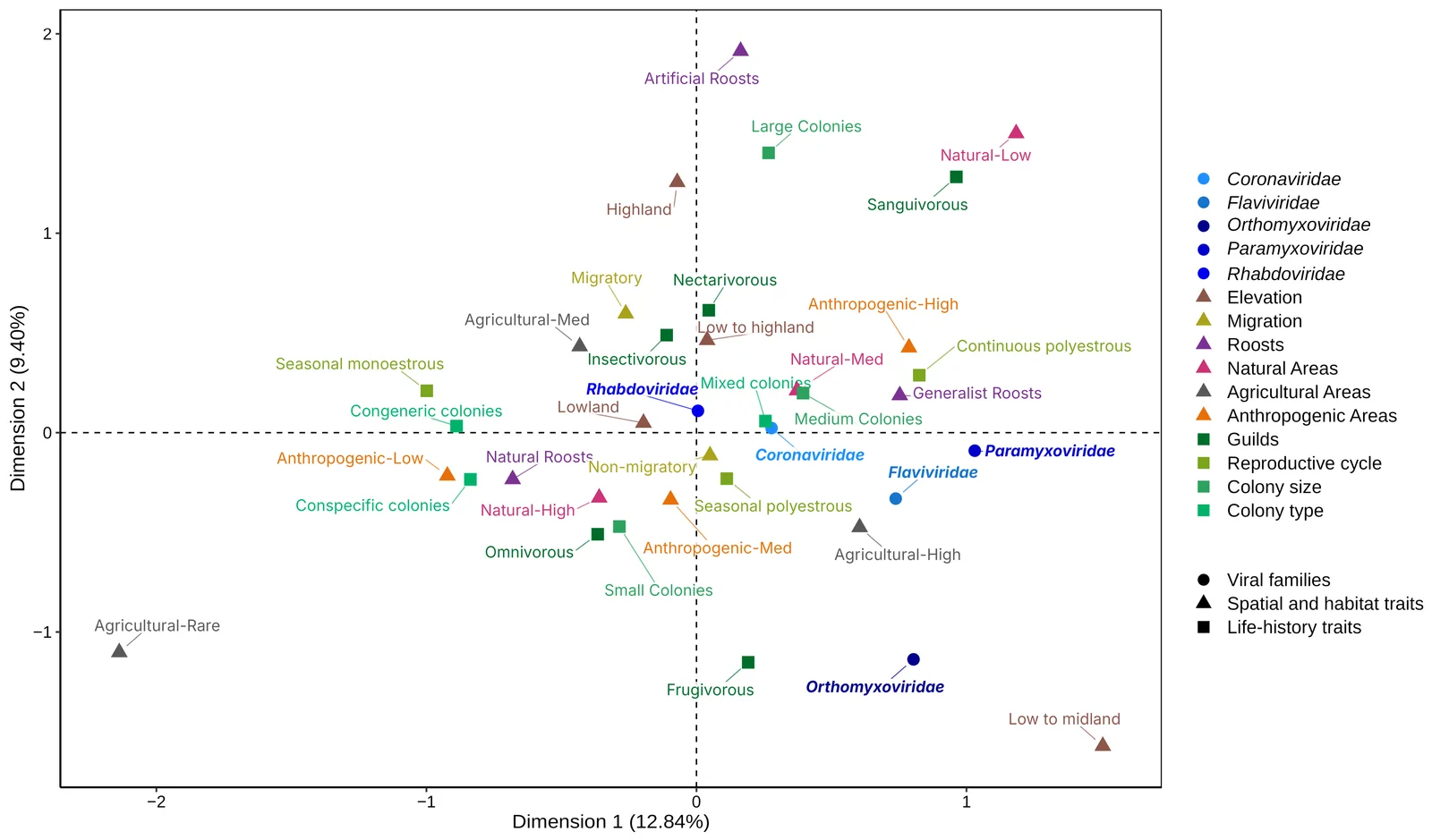
Multiple Correspondence Analysis (MCA) showing the associations between five viral families (*Coronaviridae*, *Flaviviridae*, *Orthomyxoviridae*, *Paramyxoviridae*, and *Rhabdoviridae*) and categorical variables related to spatial traits, habitat association, and life-history characteristics of bat species.

The multiple correspondence analysis (MCA) allowed us to explore the association between the detected viral families and the ecological characteristics of the host bats (Figure 4). In the multivariate space defined by the first two dimensions of the analysis (Dimension 1 = 12.84%; Dimension 2 = 9.40%; cumulative inertia = 22.23%; full eigenvalues in Table S3.1, Supplementary Material S3), four of the five viral families, *Flaviviridae*, *Paramyxoviridae*, *Orthomyxoviridae*, *and Coronaviridae*, were positioned towards positive values of Dimension 1, whereas *Rhabdoviridae* remained close to the origin of the plane. The relative proximity among the first four families in this sector of the multivariate space is consistent with shared associations with certain ecological characteristics of the hosts, although this pattern should be interpreted with caution given that the two displayed dimensions jointly capture only 22.23% of the total inertia in the dataset (Table S3.1, Supplementary Material S3).

In the factorial plane of Dimension 1, a gradient structured primarily by variables associated with anthropization, habitat use, and shelter type was evident. Toward positive values of the axis, categories related to anthropogenic environments and generalist shelters are grouped, while at the opposite extreme, conditions associated with natural habitats and specialized shelters are concentrated. The main contributors to Dimension 1 were generalist shelters (7.59%), natural shelters (6.92%), and high frequency in anthropogenic environments (7.01%), while variables such as the reproductive cycle showed a lesser contribution to the axis (full contributions for all categories are reported in Table S3.2, Supplementary Material S3).

In terms of viral families, *Paramyxoviridae* made a substantial contribution to this dimension (7.11%), followed by *Flaviviridae* (5.32%). The placement of these families toward positive values of Dimension 1 is consistent with an association with areas of high anthropogenic frequency and host species distributions ranging from low to mid-elevation.

In Dimension 2, structured patterns were primarily evidenced by variables such as foraging and colony size, meaning this dimension is mainly defined by biological and social behavioral traits like frugivorous feeding habits, which had the highest individual contribution (13.99%), followed by large colony size (11.08%) and rare presence in natural areas (7.92%). The family *Orthomyxoviridae* showed the highest contribution among the viral families to this dimension (9.09%). The multivariate analysis shows that this family notably separates towards the lower pole of Dimension 2, and was associated with frugivorous habits and host species distributed from low to medium elevation areas.

Considering the specific associations between viral families and host traits, it was evident that the *Paramyxoviridae* and *Flaviviridae* families are associated with a high frequency of occurrence in anthropogenic environments. This pattern suggests that the host species of these viruses have greater ecological plasticity or are more exposed to human-modified environments.

Regarding the family *Orthomyxoviridae*, the association observed with frugivorous habits and low-to-midland elevation suggests that diet and elevational range could be related to the distribution of this viral family. In contrast, *Coronaviridae* and *Rhabdoviridae* showed low contributions to both Dimension 1 (≤1.20%) and Dimension 2 (≤0.33%) and were positioned near the origin of the factorial plane, indicating more widespread associations and lower affinity with specific sets of ecological traits (Figure 4; Table S3.2, Supplementary Material S3).

### 3.6. Spatial and Movement Patterns

**Figure 5:**
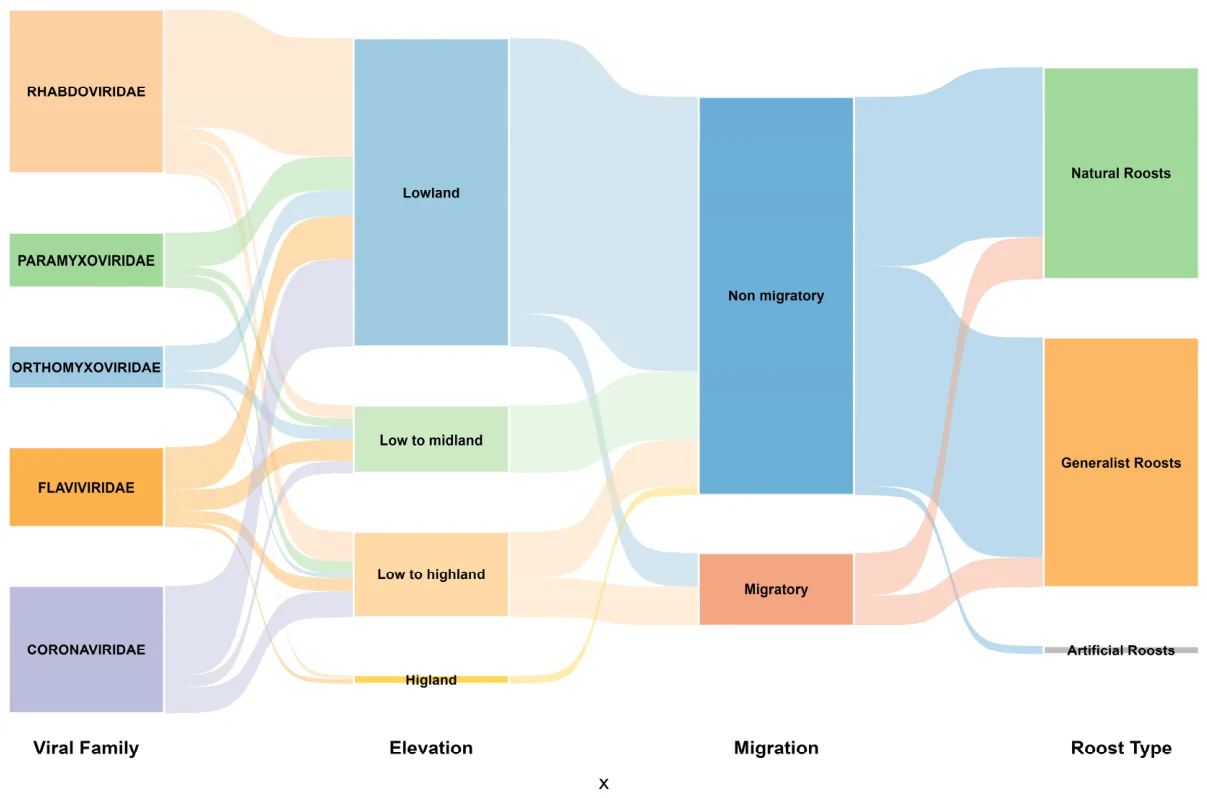
Alluvial diagram depicting the distribution and co-occurrence patterns of viral family records (*Coronaviridae*, *Flaviviridae*, *Orthomyxoviridae*, *Paramyxoviridae*, and *Rhabdoviridae*) across host spatial and movement traits, including altitudinal range, migratory behavior, and shelter type. Flow widths are proportional to the number of records represented by each category combination.

The alluvial diagrams revealed structured patterns in the distribution of the viral records across host spatial and movement attributes (Figure 5). However, statistical analyses consistently showed no significant associations between viral families and the evaluated variables (*p* > 0.05). For all three variables, more than 20% of contingency-table cells had expected frequencies below five (Table S3.3, Supplementary Material S3). Therefore, Fisher’s exact test with Monte Carlo simulation (10,000 replicates) was additionally applied, yielding conclusions consistent with those obtained using the chi-square approximation. Cramér’s V values ranged from 0.05 to 0.17, indicating weak associations across all variables. Together, these results indicate that, despite differences in record abundance among categories, no significant associations were detected between viral families and the evaluated ecological gradients.

For example, in the altitudinal distribution of host species, the alluvial diagram showed that lowland environments concentrated the largest proportion of viral records (Figure 5). Records associated with *Rhabdoviridae*, *Paramyxoviridae*, and *Orthomyxoviridae* were predominantly represented within this category. In contrast, *Coronaviridae* and *Flaviviridae* exhibited a broader distribution across elevation categories, with records occurring from low-to-midland and low-to-highland ranges. Despite the concentration of records in lowland environments, no significant association was detected between viral family and elevation (χ^2^ = 9.074, df = 12; 60% of cells with expected frequency < 5; Fisher’s exact test, simulated *p* = 0.646; V = 0.17), indicating a weak association between viral family and elevation.

Regarding migratory patterns, the alluvial diagram showed that non-migratory host species concentrated the largest proportion of viral records and included records from all analyzed viral families (Figure 5). In contrast, species exhibiting altitudinal migration represented a smaller proportion of records and were primarily associated with *Coronaviridae* and *Flaviviridae*. However, no significant association was detected between viral family and migratory behavior (χ^2^ = 0.264, df = 4; 40% of cells with expected frequency < 5; Fisher’s exact test, simulated *p* = 1.000; V = 0.05). The low Cramér’s V value indicated a very weak association between viral family and migratory behavior.

Regarding shelter use, generalist shelters (used in both natural and artificial environments) and exclusively natural shelters concentrated the largest proportion of viral records in the alluvial diagram (Figure 5). Generalist shelters included records from nearly all viral families, whereas artificial shelters represented a smaller but still notable proportion of records. Although differences in record abundance were observed among shelter categories, no significant association was detected between viral family and shelter use (χ^2^ = 5.860, df = 8; 40% of cells with expected frequency < 5; Fisher’s exact test, simulated *p* = 0.583; V = 0.16). The low Cramér’s V value indicated a weak association between viral family and shelter use.

### 3.7. Intensity of Habitat Use

**Figure 6:**
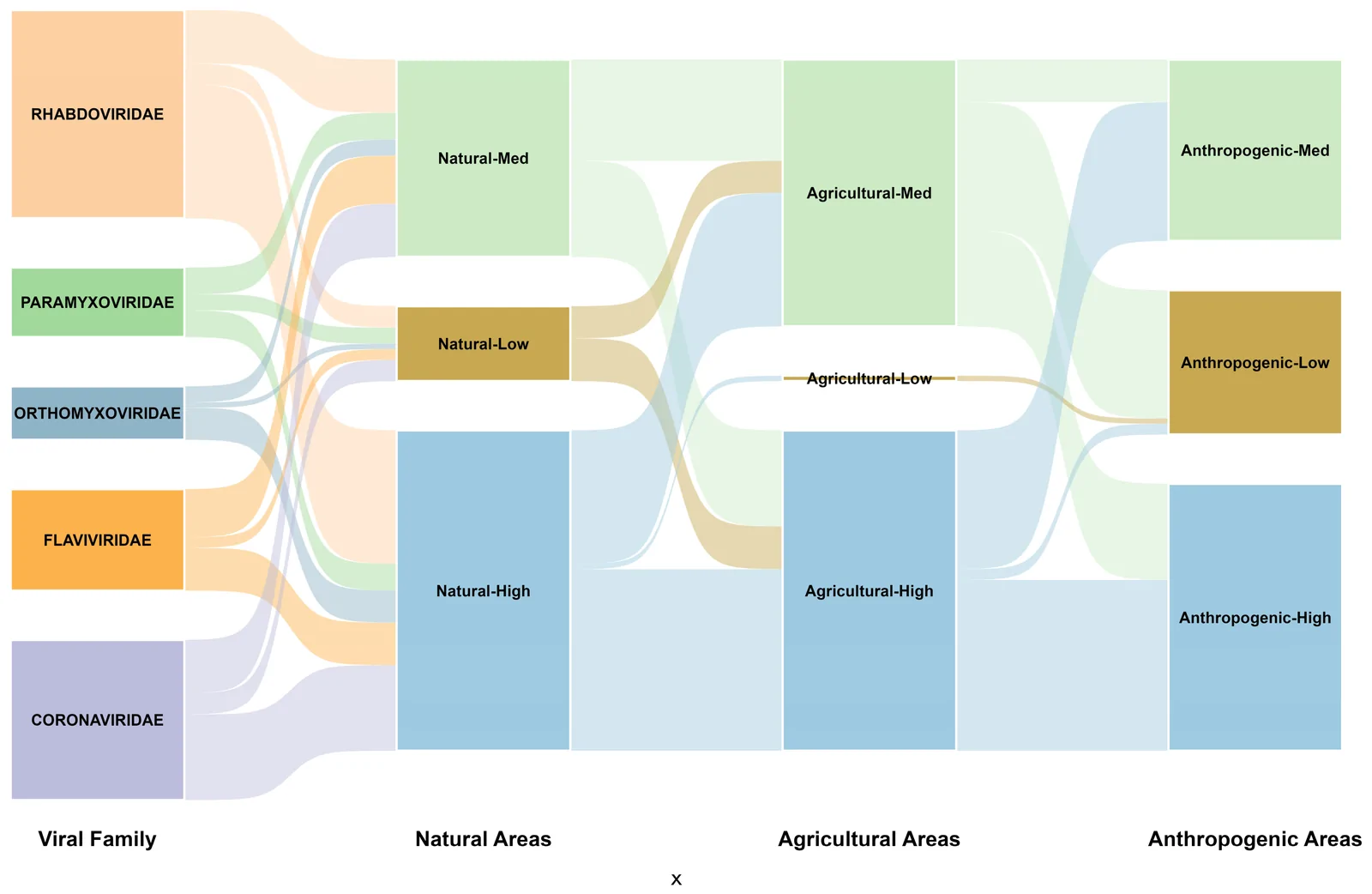
Alluvial diagram depicting the distribution and co-occurrence patterns of viral family records (*Coronaviridae*, *Flaviviridae*, *Orthomyxoviridae*, *Paramyxoviridae*, and *Rhabdoviridae*) across host habitat-use intensity categories, including natural, agricultural, and anthropogenic areas. Flow widths are proportional to the number of records represented by each category combination.

The distribution of viral family records across habitat-use categories showed patterns similar to those observed for spatial and movement traits, with differences in record abundance among categories but no statistically significant associations between viral families and habitat-use intensity (Figure 6). For the three evaluated variables (natural, agricultural, and anthropogenic areas), Fisher’s exact tests based on Monte Carlo simulation were applied because more than 20% of contingency-table cells had expected frequencies below 5 (Table S3.3, Supplementary Material S3). Results were consistent with those obtained using the chi-square approximation and remained non-significant in all cases (*p* > 0.05). Cramér’s V values ranged from 0.11 to 0.18, indicating weak association strength across habitat-use variables. Overall, viral family records were distributed across all habitat-use categories, and no significant association was detected between viral family composition and habitat-use intensity.

In natural environments, the alluvial diagram showed that viral family records were concentrated primarily in the medium- and high-use habitat categories (Figure 6). These categories included records from all evaluated viral families and were represented by the widest flows in the diagram. A similar pattern was observed in agricultural and anthropogenic environments, where medium- and high-use habitat categories concentrated the greatest proportion of records, whereas low-use categories were represented by comparatively narrower flows (Figure 6).

From a statistical perspective, no significant associations were detected between viral families and habitat-use intensity in natural, agricultural, or anthropogenic environments. In natural areas (χ^2^ = 5.267, df = 8; 46.7% of cells with expected frequency < 5; Fisher’s exact test, simulated *p* = 0.731; V = 0.15) and anthropogenic areas (χ^2^ = 2.932, df = 8; 40.0% of cells with expected frequency < 5; Fisher’s exact test, simulated *p* = 0.952; V = 0.11), weak association strengths were observed according to Cramér’s V. Similarly, in agricultural areas, where the highest Cramér’s V value among the three habitat-use variables was recorded (χ^2^ = 7.574, df = 8; 40.0% of cells with expected frequency < 5; Fisher’s exact test, simulated *p* = 0.343; V = 0.18), the association remained weak and did not reach statistical significance. These results indicate that differences in record abundance among habitat-use categories were not accompanied by significant differences in the distribution of viral families across habitat-use levels.

### 3.8. Life History Traits and Social Organization

**Figure 7:**
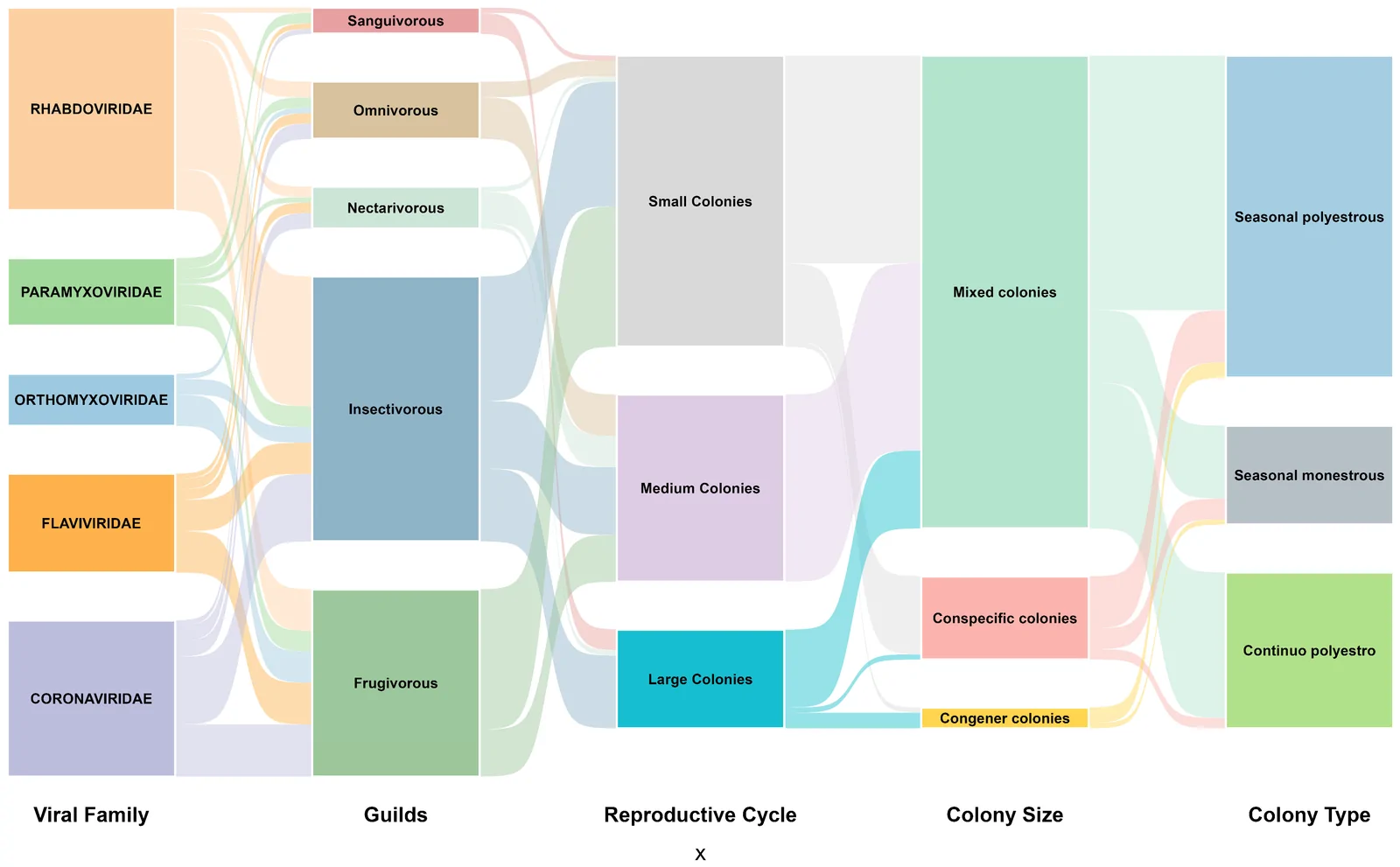
Alluvial diagram depicting the distribution and co-occurrence patterns of viral family records (*Coronaviridae*, *Flaviviridae*, *Orthomyxoviridae*, *Paramyxoviridae*, and *Rhabdoviridae*) across host natural history traits, including trophic guild, reproductive cycle, colony size, and colony type. Flow widths are proportional to the number of records represented by each category combination.

The alluvial diagrams revealed structured patterns in the distribution of viral records across host trophic guilds, reproductive cycles, colony size, and colony type (Figure 7). For all four variables, more than 20% of contingency-table cells had expected frequencies below 5 (33.3–72.0%; Table S3.3, Supplementary Material S3). Therefore, Fisher’s exact test with Monte Carlo simulation (10,000 replicates) was additionally applied, yielding conclusions consistent with those obtained using the chi-square approximation. No statistically significant associations were detected between viral families and any of the evaluated traits (*p* > 0.05 in all cases). Cramér’s V values ranged from 0.13 to 0.20, indicating weak associations across all variables.

For trophic guilds, the alluvial diagram showed that insectivorous and frugivorous bats concentrated the largest proportion of viral records and included records from all analyzed viral families (Figure 7). However, no significant association was detected between viral family and trophic guild (χ^2^ = 15.997, df = 16; 72.0% of cells with expected frequency < 5; Fisher’s exact test, simulated *p* = 0.405; V = 0.19), indicating a weak association between viral family composition and host feeding guild.

Regarding reproductive cycles and colony size, viral records were primarily concentrated in hosts exhibiting seasonal polyestrous reproductive cycles and in small- to medium-sized colonies (Figure 7). However, neither reproductive cycle (χ^2^ = 9.32, df = 8; 33.3% of cells with expected frequency < 5; Fisher’s exact test, simulated *p* = 0.289; V = 0.20) nor colony size (χ^2^ = 3.84, df = 8; 33.3% of cells with expected frequency < 5; Fisher’s exact test, simulated *p* = 0.889; V = 0.13) showed a significant association with viral family. In both cases, Cramér’s V values indicated weak association strength.

Finally, the alluvial diagram showed that mixed colonies concentrated the largest proportion of viral records among colony types, followed by conspecific and congeneric colonies (Figure 7). Despite differences in record abundance among colony categories, no significant association was detected between viral family and colony type (χ^2^ = 4.06, df = 8; 60.0% of cells with expected frequency < 5; Fisher’s exact test, simulated *p* = 0.854; V = 0.14). The low Cramér’s V value indicated a weak association between viral family composition and colony type.

## 4. Discussion

### 4.1. The Eco-Virological Scenario of the Northern Andes

The present research represents a comprehensive effort to characterize potential eco-virological patterns associated with bat species occurring in the Northern Andes, based on 578 unique viral records documented across the Neotropics for 56 bat species whose geographic distributions include this region (Supplementary Material S1). This region is part of the Tropical Andes, recognized as the richest biodiversity hotspot on the planet [59,60,61], harboring approximately 10% of all mammal species in an area covering just 1% of the Earth’s land surface [60,62,63].

Mammals represent one of the most diverse groups, with a notable richness of chiropterans exceeding 260 species in the northern region [7,64,65]. This diversity is predominantly concentrated in the low foothills and premontane forests (below 2100 m above sea level) [66,67], where the greater availability of seasonal resources and habitat connectivity not only favor diverse communities but also facilitate the persistence and exchange of viral taxa among populations [6,10,68]. Such biogeographic characteristics consolidate the region as a pool of massive and diverse viruses, making it a critical area for the surveillance of emerging zoonoses globally.

The epidemiological importance of this hotspot is accentuated by the demographic configuration of the northern Andes, which extends from the Barquisimeto-Carora depression in Venezuela to the Gulf of Guayaquil in Ecuador [69,70]. This region supports one of the highest human densities on the continent: the Colombian Andean region is home to 34.4 million people (which is the most active economic zone in the country), while in Ecuador between 8 and 9 million inhabitants reside in the Andean highlands, and the Venezuelan Andes concentrate another 6 to 7 million people [71,72,73]. This confluence generates a massive contact interface between humans, domestic animals, and wild reservoirs, especially in landscapes where deforestation already exceeds 60% of the original coverage [74,75].

However, despite these conditions that could favor wildlife–human interactions, the available evidence for the Northern Andes remains remarkably limited. Most viral records included in this review originated from Brazil (64%), whereas Colombia represented only 5% of the dataset and no records were identified for Ecuador or Venezuela. Consequently, the patterns described here should be interpreted as a proxy derived from Neotropical records of bat species occurring in the Northern Andes rather than direct evidence of viral circulation within the region itself. Nevertheless, this information gap is particularly relevant in a territory inhabited by approximately 48–50 million people, where the absence of local surveillance may hinder the identification of zoonotic risks and lead to an underestimation of potential spillover processes involving viruses that could be associated with bat species present in the region.

On the other hand, the Phyllostomidae family emerges as the most represented primary reservoir, accounting for 60.9% of the records (Figure 3) and being the only one present in the five viral families analyzed (Table 1). While this figure is consistent with its status as the most diverse and abundant family of Neotropical bats, it is also likely to reflect a sampling or detection bias inherent to the available literature, which tends to focus on species that are easier to capture or have a recurring presence in diverse habitats [76]. Nevertheless, its documented ecological traits suggest that it may play a relevant role as an epidemiological bridge, as the high mobility and ecological plasticity allow many Phyllostomidae species, especially generalists, to adapt and thrive in environments transformed by the expansion of the agricultural frontier [76,77,78].

Another notable finding in this review is the profound asymmetry between the volume of records and taxonomic diversity. The viral family *Rhabdoviridae*, despite leading with 352 records, was exclusively restricted to the rabies virus (*Lyssavirus rabies*), highlighting a historical surveillance bias in the Neotropics, primarily focused on the economic impact of rabies in livestock [27,79,80,81]. Additionally, 19.4% of the total dataset corresponded to records documented in *Desmodus rotundus* (Supplementary Material S1), consolidating this species as the primary hematophagous reservoir implicated in the maintenance of the rabies cycle [82]. This high representativeness is consistent with its ecological plasticity and foraging habits, which favor close contact with livestock in landscapes where habitat fragmentation and deforestation have altered their natural refuge sites, facilitating the persistence and spread of viruses at the human–animal interface [83].

Regarding the family *Coronaviridae*, its representativeness was marked by the dominance of the genus *Alphacoronavirus*, which accounted for 74% of the records (Table 2). This finding is consistent with the literature associating a higher detection of coronaviruses in generalist species compared to specialists [84,85,86]. In human-modified landscapes across the Neotropics, including areas within the Northern Andes, habitat fragmentation and the expansion of urban and rural settlements have created new contact interfaces; the ability of these hosts to occupy anthropogenic shelters may indicate a viral maintenance dynamic favored by such conditions. Therefore, constant interaction in transformed landscapes could increase opportunities for interspecific transmission, suggesting alphacoronaviruses as a relevant taxon for surveillance in the face of emerging virus risks [87].

In contrast, the family *Flaviviridae* exhibited the highest taxonomic richness with seven identified taxa, including viruses of high public health impact such as dengue and zika (Table 2). Although these records originate from diverse regions of the Neotropics, their identification allows for the inference of a potential risk scenario for the Northern Andes, based on the geographic distribution of host species that are also known to occur in the region.

This inferred risk scenario is highly consistent with the endemicity of the Andean and sub-Andean regions, where thermal and elevation thresholds allow the persistence of these arboviruses up to critical limits such as 2100 m above sea level, with vector populations declining sharply above 1500–1700 m as temperature decreases [88,89], primarily conditioned by the altitudinal distribution of their vectors (e.g., *Aedes aegypti*, *Ae. albopictus*), whose presence is regulated by thermal restrictions that affect their survival, population dynamics, and viral transmission efficiency [90,91].

On the other hand, although *Paramyxoviridae* and *Orthomyxoviridae* were the least frequently reported families in this review, both included viral taxa of recognized zoonotic interest. Within *Paramyxoviridae*, zoonotic genera such as *Morbillivirus* and *Parajeilongvirus* were identified [28,92,93], whereas within *Orthomyxoviridae*, bat-associated influenza A virus subtypes such as H17N10 and H18N11 have been reported in the literature [94,95]. Although these taxa were recorded less frequently than those of other viral families, their occurrence expands the diversity of viral lineages documented in Neotropical bats. In regions characterized by high biodiversity and frequent human–wildlife interfaces, including the Northern Andes, these observations highlight the importance of continued surveillance efforts aimed at improving our understanding of host–virus associations under scenarios of agricultural intensification and biodiversity loss [1,6].

In summary, the marked information gap identified for the Northern Andes highlights the need to strengthen virological surveillance in bat communities throughout the region. The predominance of records from a limited number of host species and viral taxa suggests that current knowledge remains incomplete and potentially biased toward the best-studied systems. Nevertheless, the detection of zoonotic viral groups such as *Alphacoronavirus*, *Orthoflavivirus*, *Morbillivirus*, and Influenza A in bat species whose distributions include the Northern Andes underscores the value of these hosts as priorities for future surveillance efforts.

Expanding systematic monitoring under a One Health framework that integrates wildlife, environmental, and public health perspectives [14,17] would improve our understanding of potential host–virus associations and help reduce critical regional knowledge gaps.

### 4.2. Ecological Patterns of Viral Records Across Potential Bat Host Species

*Spatial and movement patterns*—The MCA restricted to elevation, migratory behavior, and roost type revealed that *Orthomyxoviridae* was the family showing the clearest separation within the ordination space, being positioned toward the negative extreme of Dimension 2 and in close association with the “low to mid elevation” category. This pattern is consistent with the fact that several bat species reported as hosts of this viral family are predominantly distributed across low and intermediate elevational ranges [96,97]. Such a distribution agrees with broader patterns of bat species richness along Neotropical elevational gradients, where diversity is generally concentrated at lower elevations and declines progressively with increasing altitude [98]. Therefore, the signal observed for *Orthomyxoviridae* may reflect, at least in part, the elevational distribution of the host species reported for this family rather than an intrinsic ecological preference of the virus itself.

In contrast, *Paramyxoviridae* and *Flaviviridae* clustered toward the positive pole of Dimension 1, in proximity to the “generalist roost use” category. This configuration is consistent with previous reports indicating that several host species associated with these viral families exploit a wide variety of roost types, including both natural structures and habitats modified by human activities [39,40]. Such a pattern aligns with evidence suggesting that species exhibiting generalist roosting behavior experience increased opportunities for intra- and interspecific interactions, a characteristic that has been proposed as relevant to viral maintenance and detection in diverse bat populations [99]. In contrast, *Rhabdoviridae* and *Coronaviridae* remained close to the origin of the ordination plot, indicating limited differentiation with respect to the categories considered in this analysis. Likewise, none of the five viral families displayed a clear separation along the gradient associated with migratory behavior, suggesting that this variable contributed little to the structure observed in the factorial space.

These patterns were broadly consistent with the trajectories observed in the alluvial diagram (Figure 5). Overall, the flows showed a marked concentration of records within the categories corresponding to low elevations, non-migratory species, and natural or generalist roosts, regardless of viral family. While migratory behavior contributed little to the differentiation of viral families in the ordination, the predominance of records associated with resident, non-migratory species is consistent with the hypothesis that spatially stable host populations may act as persistent reservoirs, favoring the long-term maintenance of host–virus associations at local scales [100]. This interpretation should nonetheless be regarded as speculative, as spatial stability alone does not establish a causal relationship between migratory behavior and viral occurrence.

*Habitat use intensity*—From an eco-epidemiological perspective, the MCA restricted to habitat-use intensity variables suggested that differences among viral families were associated more closely with the way host species use natural, agricultural, and anthropogenic environments than with any single habitat type. *Paramyxoviridae*, *Flaviviridae*, and *Orthomyxoviridae* grouped toward the positive pole of Dimension 1, in proximity to the categories “Anthropogenic High,” “Agricultural High,” and “Natural Low,” whereas “Anthropogenic Low,” “Agricultural Medium,” and “Natural High” were positioned toward the opposite pole. This configuration suggests that records associated with these viral families were more frequently linked to host species reported to make intensive use of managed landscapes and comparatively lower use of natural habitats [101,102]. Along Dimension 2, *Orthomyxoviridae* occupied a more distinct position, particularly in relation to “Agricultural-High,” whereas *Rhabdoviridae* and *Coronaviridae* remained close to the origin of the ordination space, indicating limited differentiation with respect to the habitat-use categories considered.

These patterns were broadly consistent with the alluvial diagram (Figure 6), where records flowed predominantly through high-intensity categories across natural, agricultural, and anthropogenic environments. Rather than indicating dependence on a particular habitat type, this distribution suggests that many host species associated with the analyzed viral families are capable of exploiting multiple landscape types, including human-modified environments [15,17].

This interpretation is consistent with ecological information reported elsewhere in this review. Several bat species associated with multiple viral families, including *Carollia perspicillata*, *Glossophaga soricina*, and *Artibeus lituratus* (Supplementary Material S2), have been documented occupying both natural habitats and disturbed or human-modified environments [100]. Such ecological flexibility has been proposed as a characteristic that may increase opportunities for contact among hosts across heterogeneous landscapes [103]. Nevertheless, these observations should be interpreted as descriptive and hypothesis-generating, providing ecological context for the grouping patterns identified by the MCA and the alluvial diagram rather than evidence of causal relationships between habitat use and viral occurrence.

*Life history traits and social organization*—The MCA restricted to diet, reproductive cycle, colony size, and colony type revealed that Dimension 2 was primarily structured by the frugivorous guild, which was positioned in close proximity to *Orthomyxoviridae*. This pattern suggests that records associated with this viral family were more frequently reported in frugivorous host species. However, this association should be interpreted cautiously, as frugivorous and insectivorous guilds also represent the most diverse and abundant host groups included in this review [104,105], which may partly explain the observed pattern. Nevertheless, previous studies have reported higher viral prevalence in ecologically flexible frugivorous species, such as *Artibeus* spp. and *Carollia* spp., particularly in fragmented landscapes [106,107], whereas other authors have attributed patterns of viral exposure to specific dietary traits rather than to frugivory itself [108]. Because the present analysis does not allow these alternative explanations to be disentangled, the observed pattern is interpreted as being consistent with both possibilities rather than as evidence of a particular biological affinity between *Orthomyxoviridae* and frugivorous bats.

Along Dimension 1, reproductive cycle contributed more substantially to the ordination than colony size. The “continuous polyestrous” category showed the strongest alignment with viral family records, whereas “seasonal monoestrous” occupied the opposite pole of the axis. Although this study did not directly evaluate the relationship between reproduction, immunity, and viral dynamics, this pattern is consistent with the hypothesis that hosts exhibiting more frequent reproductive cycles may sustain a more continuous recruitment of immunologically naïve individuals throughout the year, potentially creating more persistent opportunities for viral maintenance within host populations, in contrast to the more temporally concentrated recruitment expected under a strictly monoestrous reproductive strategy [107,109].

In contrast, medium-sized colonies (50–500 individuals) were located near the origin of the ordination space and contributed minimally to either dimension, suggesting that colony size was not a major axis of differentiation in this exploratory analysis. This observation contrasts with classical density-dependent transmission models, which predict that larger colonies should facilitate higher transmission rates through increased contact opportunities [110,111]. However, it is consistent with the findings of Bergner et al. [109], who likewise found no evidence that larger colonies of *D. rotundus* harbor greater viral richness. This discrepancy may reflect, among other factors, biases associated with the available literature or differences among the ecological systems being compared. Nevertheless, these explanations should be regarded as hypotheses requiring targeted evaluation rather than inferences directly supported by the data presented here.

These patterns were broadly reflected in the alluvial diagram (Figure 7), where records were concentrated primarily within the insectivorous and frugivorous guilds, small and mixed colonies, and polyestrous reproductive strategies. Although mixed colonies contributed little to the MCA ordination, they accounted for the largest volume of records in the alluvial diagram across the five viral families. While descriptive, this pattern is noteworthy in light of recent evidence suggesting that the species composition of shared roosts may influence opportunities for viral exchange within sympatric bat communities [112]. Taken together, the MCA and alluvial results suggest that colony composition and reproductive frequency may represent informative variables for future hypothesis-driven research, rather than confirmed drivers of viral transmission within the dataset analyzed here.

### 4.3. Statistical Significance and Interpretive Limitations of the Exploratory Analyses

Across the three dimensions examined above—spatial and movement patterns, habitat use intensity, and life-history and social traits—no statistically significant associations were detected between viral families and the ecological, biological, and spatial traits evaluated, with consistently low Cramér’s V values (0.05–0.20). These findings should be interpreted cautiously, as non-significant results do not necessarily indicate the absence of ecological structuring or demonstrate a uniform distribution of viral families across host traits. Given the moderate sample size (*n* = 56 species) and the low effect sizes observed, the absence of statistically significant associations may partly reflect limited statistical power (Type II error) [113] rather than a truly homogeneous distribution of viral families across ecological dimensions [114,115].

To further evaluate this possibility, 95% confidence intervals for Cramér’s V were calculated for each test (Table S3.3, Supplementary Material S3); upper bounds reached up to 0.27, indicating that the data cannot rule out associations of small-to-moderate magnitude and reinforcing the plausibility of a Type II error rather than a true absence of association. Nevertheless, even under this scenario, the alluvial diagrams show that records of each viral family are distributed across multiple, ecologically distinct host categories rather than concentrated within a single profile. As a working hypothesis, this pattern may reflect a form of viral transversality [116], herein defined as the occurrence of records from a given viral family across host species that differ in their ecological traits, rather than being restricted to a particular ecological profile.

As a conceptual framework, the hypothesis of viral transversality proposed here is broadly consistent with the pathogen pool hypothesis, which proposes that highly diverse ecosystems can maintain extensive pathogen reservoirs whose circulation is not necessarily constrained by narrow ecological associations [117]. Although the present dataset does not allow this pattern to be directly evaluated for the Northern Andes, the combination of high species diversity and increasing landscape transformation in Andean and sub-Andean environments [118,119] provides a context in which similar dynamics may occur.

However, both this exploratory interpretation and the broader pattern of non-significant associations described above should be treated with caution, as they may reflect one or a combination of several non-mutually exclusive methodological factors: (i) limited statistical power associated with the moderate sample size and low effect sizes observed; (ii) heterogeneity in sampling effort among studies and regions, which may influence the apparent distribution of viral records [120]; and/or (iii) phylogenetic non-independence among host species, whereby closely related taxa share ecological and evolutionary characteristics that can influence viral occurrence patterns [121].

In conclusion, this review represents a pioneering effort to synthesize records of potentially zoonotic RNA viruses reported across the Neotropics for bat species whose geographic distributions include the Northern Andes. By integrating viral detection records with ecological, spatial, and life-history traits, our study provides a preliminary framework for exploring host–virus associations relevant to bat assemblages occurring in this region. The predominance of records associated with Phyllostomidae and the strong representation of rabies-related reports highlight important patterns in the currently available literature, while also revealing potential sampling biases. At the same time, the marked scarcity of records from Colombia and the absence of records from Ecuador and Venezuela expose a critical regional knowledge gap that limits our understanding of viral diversity and host–virus associations in the Northern Andes. Furthermore, only a fraction of the region’s bat diversity has been investigated, and in many cases, viral detections are not resolved beyond the family or genus level—either because the original studies did not report species-level identification or because the virus itself remains taxonomically undescribed—nor is the clinical or pathological status of infected animals consistently reported, constraining future predictive analyses of viral dynamics [108].

It is equally important to note that the same traits that make bats notable hosts of diverse viruses do not by themselves imply an elevated or indiscriminate risk to human health. Bats possess a distinctive immune tolerance, shaped by long evolutionary adaptation to flight, that allows many viral infections to persist without causing overt disease in the host [14]. In addition, their nocturnal and cryptic habits generally limit direct contact with humans, meaning that spillover events typically require additional ecological, behavioral, or anthropogenic conditions to occur, rather than resulting automatically from the presence of a virus in a bat population. Rather than representing only a source of risk, bats constitute valuable subjects for epidemiological and environmental surveillance programs, given their broad taxonomic and geographic diversity and their sensitivity to landscape change [122].

Consequently, this study underscores the urgent need to strengthen virological surveillance and ecological monitoring of bat communities in the Northern Andes under a One Health framework that integrates wildlife, environmental, and public health perspectives to reduce current knowledge gaps and improve preparedness for future zoonotic threats.

## Figures and Tables

**Table 1:** Number of viral records by bat family and viral family. Abbreviations: CoV, *Coronaviridae*; Flavivi, *Flaviviridae*; Orthomx, *Orthomyxoviridae*; Pmxv, *Paramyxoviridae*; Rhabdo, *Rhabdoviridae*.

Host Family	CoV	Flavivi	Othomx	Pmxv	Rhabdo	No. Records
Phyllostomidae	107	36	13	30	166	352
Molossidae	20	5	—	2	91	118
Vespertilionidae	6	1	—	1	93	101
Noctilionidae	2	—	1	—	1	4
Mormoopidae	1	—	—	—	1	2
Emballonuridae	—	1	—	—	—	1
No. Records	136	43	14	33	352	578

Note: A dash (—) indicates that no viral records were identified for the corresponding bat family–viral family combination.

**Table 2:** Taxonomic composition and resolution of viral records by family.

*Coronaviridae*	*Flaviviridae*	*Orthomyxoviridae*	*Paramyxoviridae*	*Rhabdoviridae*
*Coronaviridae* unclassified *n* = 3	*Flaviviridae* unclassified *n* = 4	Influenza A virus *n* = 10	*Paramyxoviridae* unclassified *n* = 14	*Lyssavirus rabies* *n* = 352
*Coronavirinae* unclassified *n* = 23	*Flavivirus* unclassified *n* = 4	Influenza A virus (H17N10) *n* = 2	*Morbillivirus* *n* = 10	
*Unclassified Orthocoronavirinae* *n* = 1	*Hepacivirus* *n* = 3	Influenza A virus (H18N11) *n* = 2	*Orthorubulavirus suis* **n* = 1*	
*Alphacoronavirus* *n* = 101	*Bovine Hepacivirus* *n* = 1		*Parajeilongvirus* *n* = 2	
*Betacoronavirus* *n* = 8	*Orthoflavivirus denguei* *n* = 16		*Parajeilongvirus carolliae* *n* = 2	
	*Orthoflavivirus zikaense* *n* = 5		*Parajeilongvirus hainanense* *n* = 4	
	*Pegivirus* (bat) *n* = 10			

## Data Availability

The data supporting the findings of this study are fully available in the Supplementary Materials accompanying this article. The scoping review was registered at https://doi.org/10.17605/OSF.IO/U5MGA. The viral sequence accession numbers compiled from the reviewed literature are publicly available in the NCBI Nucleotide database, as originally deposited by their respective authors.

## Supplementary Materials

The following supporting information can be downloaded at https://www.mdpi.com/article/10.3390/v18101148/s1: Supplementary Material S1: Neotropical dataset; Supplementary Material S2: Ecology analysis; Supplementary Material S3: Statistical results.
