# Supplementary material for: Eco-Virological Patterns of Zoonotic Viruses in Neotropical Bats: Insights for Host Species Distributed in the Northern Andes—A Scoping Review and Trait-Based Analysis
Source: Viruses. 2026 Oct 16;18(10):1148. doi: 10.3390/v18101148 (PMC13709678; doi:10.3390/v18101148)
Supplement: Supplementary file 1 [file viruses-18-01148-s001.zip › Supplementary_Material_S3 Statistical results.pdf]

Supplementary Material S3: Statistical Results

Multiple Correspondence Analysis (MCA) and Chi-Square Tests of Independence between viral families and host ecological traits.

- Multiple Correspondence Analysis (MCA).

Table S3.1. Eigenvalues and percentage of inertia explained by each dimension of the Multiple Correspondence Analysis (MCA). The MCA was performed on 15 categorical variables (5 viral families and 10 host ecological traits) recorded for 56 bat species, yielding 27 dimensions. The first two dimensions, used for graphical representation in Figure 4, accounted jointly for 22.23% of the total inertia (highlighted). The cumulative percentage of inertia explained by successive dimensions is also shown.

Dimension	Eigenvalue	% of variance (inertia)	Cumulative %
**Dim 1**	**0.231**	**12.84%**	**12.84%**
**Dim 2**	**0.169**	**9.40%**	**22.23%**
Dim 3	0.139	7.75%	29.98%
Dim 4	0.131	7.28%	37.26%
Dim 5	0.119	6.64%	43.90%
Dim 6	0.110	6.12%	50.02%
Dim 7	0.106	5.90%	55.92%
Dim 8	0.097	5.36%	61.29%
Dim 9	0.084	4.65%	65.94%
Dim 10	0.070	3.87%	69.81%
Dim 11	0.066	3.67%	73.49%
Dim 12	0.064	3.57%	77.05%
Dim 13	0.057	3.14%	80.20%
Dim 14	0.048	2.69%	82.89%
Dim 15	0.047	2.60%	85.49%
Dim 16	0.042	2.33%	87.81%
Dim 17	0.032	1.80%	89.61%
Dim 18	0.031	1.73%	91.34%
Dim 19	0.029	1.58%	92.93%
Dim 20	0.028	1.56%	94.48%
Dim 21	0.023	1.26%	95.74%
Dim 22	0.022	1.20%	96.94%
Dim 23	0.017	0.95%	97.89%
Dim 24	0.013	0.73%	98.62%
Dim 25	0.010	0.57%	99.19%
Dim 26	0.008	0.45%	99.64%
Dim 27	0.007	0.36%	100.00%

Table S3.2. Coordinates, contributions, and squared cosine values (cos^2) of MCA categories on Dimensions 1 and 2. Contributions (%) indicate the relative importance of each category in defining the corresponding dimension, whereas cos^2 values represent the quality of representation of each category on the factorial plane. Categories are ordered according to their contribution to Dimension 1. Categories labeled “Non” (absence of a given viral family) were included as supplementary (passive) categories in the MCA and are reported here together with the active categories for completeness.

Category	Dim1 coord.	Dim2 coord.	Dim1 contrib. (%)	Dim2 contrib. (%)	Dim1 cos^2	Dim2 cos^2
Generalist Roosts	0.753	0.187	7.59	0.64	0.491	0.030
Anthropogenic-Low	-0.923	-0.216	7.45	0.56	0.371	0.020
Seasonal monestrous	-0.999	0.210	7.19	0.43	0.332	0.015
PARAMYXOVIRIDAE	1.031	-0.090	7.11	0.07	0.321	0.002
Anthropogenic-High	0.787	0.426	7.02	2.81	0.401	0.118
Natural Roosts	-0.681	-0.234	6.92	1.12	0.498	0.059
Low to midland	1.505	-1.570	5.83	8.67	0.222	0.242
FLAVIVIRIDAE	0.738	-0.331	5.33	1.46	0.280	0.056
Agricultural-High	0.604	-0.475	4.70	3.96	0.295	0.182
Continuo polyestro	0.825	0.288	4.56	0.76	0.206	0.025
Conspecific colonies	-0.837	-0.234	3.97	0.43	0.171	0.013
Natural-Low	1.184	1.501	3.61	7.93	0.137	0.221
ORTHOMYXOVIRIDAE	0.804	-1.137	3.33	9.09	0.140	0.281
Agricultural-Med	-0.432	0.432	2.89	3.94	0.216	0.216
Non-FLAVIVIRIDAE	-0.379	0.170	2.74	0.75	0.280	0.056
Agricultural-Rare	-2.137	-1.102	2.35	0.85	0.083	0.022
Natural-High	-0.360	-0.326	2.27	2.55	0.200	0.165
Non-PARAMYXOVIRIDAE	-0.312	0.027	2.15	0.02	0.321	0.002
Mixed colonies	0.255	0.058	1.45	0.10	0.216	0.011
Non-CORONAVIRIDAE	-0.321	-0.027	1.38	0.01	0.089	0.001
Medium Colonies	0.395	0.198	1.37	0.47	0.068	0.017
Small Colonies	-0.285	-0.471	1.30	4.84	0.101	0.275
Natural-Med	0.372	0.211	1.21	0.53	0.060	0.019
CORONAVIRIDAE	0.278	0.023	1.20	0.01	0.089	0.001
Sanguivorous	0.962	1.282	0.95	2.31	0.034	0.061
Congener colonies	-0.889	0.033	0.81	0.00	0.029	0.000
Lowland	-0.196	0.049	0.79	0.07	0.096	0.006
Non-Orthomyxo	-0.175	0.247	0.72	1.98	0.140	0.281
Omnivorous	-0.366	-0.509	0.34	0.91	0.013	0.025
Migratory	-0.262	0.597	0.32	2.26	0.013	0.068
Large Colonies	0.267	1.403	0.29	11.08	0.012	0.328
Frugivorous	0.192	-1.151	0.28	14.00	0.013	0.485
Seasonal polyestrous	0.112	-0.230	0.19	1.08	0.014	0.057
Insectivorous	-0.110	0.489	0.18	4.88	0.013	0.257
Anthropogenic-Med	-0.096	-0.336	0.08	1.35	0.004	0.049
Non migratory	0.050	-0.114	0.06	0.43	0.013	0.068
Low to highland	0.039	0.464	0.01	1.51	0.000	0.047
Artificial Roosts	0.163	1.914	0.01	2.58	0.000	0.067
Nectarivorous	0.046	0.614	0.01	1.33	0.000	0.037

Category	Dim1 coord.	Dim2 coord.	Dim1 contrib. (%)	Dim2 contrib. (%)	Dim1 cos²	Dim2 cos²
Non-RHABDOVIRIDAE	-0.013	-0.251	0.00	0.75	0.000	0.027
RHABDOVIRIDAE	0.006	0.109	0.00	0.33	0.000	0.027
Higland	-0.071	1.256	0.00	1.11	0.000	0.029

- ## Chi-square association tests

Table S3.3. Chi-square tests of independence and Cramér's V effect sizes evaluating associations between viral families and host ecological traits. For each contingency table, the assumption that expected cell frequencies were ≥5 was assessed. When more than 20% of cells had expected frequencies <5, Fisher's exact test with Monte Carlo simulation (10,000 replicates) was used in place of the chi-square approximation (see Materials and Methods). Statistical significance was evaluated at α = 0.05. No statistically significant associations were detected for any of the traits analyzed.

Category	Variable	χ^2	df	p (χ^2)	% cells < 5	Test used	p (final)	Cramér's V	95% CI
Spatial and movement patterns	Elevation	9.07	12	0.697	60.0%	Fisher (sim., B=10,000)	0.646	0.165	[0.000, 0.175]
	Migration	0.26	4	0.992	40.0%	Fisher (sim., B=10,000)	1.000	0.049	[0.000, 0.000]
	Roost type	5.86	8	0.663	40.0%	Fisher (sim., B=10,000)	0.583	0.163	[0.000, 0.207]
Intensity of habitat use	Natural Areas	5.27	8	0.729	46.7%	Fisher (sim., B=10,000)	0.731	0.154	[0.000, 0.193]
	Agricultural Areas	7.57	8	0.476	40.0%	Fisher (sim., B=10,000)	0.343	0.185	[0.000, 0.241]
	Anthropogenic Areas	2.93	8	0.939	40.0%	Fisher (sim., B=10,000)	0.952	0.115	[0.000, 0.105]
Life history & social organization	Guilds	16.00	16	0.453	72.0%	Fisher (sim., B=10,000)	0.405	0.190	[0.000, 0.195]
	Reproductive cycle	9.32	8	0.316	33.3%	Fisher (sim., B=10,000)	0.289	0.205	[0.000, 0.269]
	Colony size	3.85	8	0.871	33.3%	Fisher (sim., B=10,000)	0.889	0.132	[0.000, 0.150]
	Colony type	4.06	8	0.851	60.0%	Fisher (sim., B=10,000)	0.854	0.135	[0.000, 0.157]

Abbreviations: χ^2, chi-square statistic; df, degrees of freedom; p (χ^2), p-value from the chi-square test; % cells < 5, percentage of contingency-table cells with expected frequencies <5; Test used, inferential test retained for that variable; p (final), p-value from the test retained for inference (chi-square or Fisher's exact test, as indicated); Cramér's V, effect-size measure of association; 95% CI, 95% confidence interval for Cramér's V.
